# The Health of the Clergy

**Published:** 1844-10

**Authors:** 


### THE HEALTH OF THE CLERGY.

A premium of twenty dollars for the best essay, on the "*Importance of good health in ministers of the gospel, and the proper means of securing it,*" is offered by "a Lady," in the Protestant and Herald newspaper. The sum has been placed in the hands of the Rev. W. L. Breckinridge, who with the Rev. E. P. Humphrey, and Dr. L. P. Yandell, is designated by the fair giver to judge of the essays that may be offered, and award the prize. Mr. Breckinridge has given notice through the same paper, that the committee "will receive any communications which may be offered, free of cost, until after the first of January next, immediately after which the premium will be awarded to the one deemed most worthy of it."

A prize held out by such a hand, for such an object, ought to bring forth a crowd of competitors.

